# Society Meetings

**Published:** 1903-03

**Authors:** 


### SOCIETY MEETINGS.

---

The Fourteenth International Medical Congress will be held this year in Madrid, from April 23 to 30, and as usual a number of physicians accompanied by their families are planning to attend the meeting.

It is therefore thought that it would be of advantage to arrange a number of conducted tours from this country to and from Madrid, as in this way better accommodations can be obtained at reduced prices, besides which, those participating can choose the routes which will enable them to visit the different parts of the Continent that they are desirous of seeing. For this reason four routes have been planned. For particulars address Dr. Ramon Guiteras, 75 W. 55th Street, New York, or Dr. Charles Wood Fassett, St. Joseph, Mo.

---

The Western Ophthalmologic and Oto-laryngologic Association will hold its eighth annual meeting at Indianapolis, Ind., April 9, 10 and 11, 1903. Dr. William L. Ballenger, of Chicago, is the president, and Dr. Derrick T. Vail, of Cincinnati, is the secretary.

---

The Buffalo Academy of Medicine held meetings during the months of January and February as follows:

*Section on Obstetrics and Gynecology.*—Tuesday evening, January 27. Program: Diagnosis of exceptional conditions in labor, Stephen Y. Howell. Report of anomalous cases, Henry G. Bentz. Ancient gynecology, Henry D. Ingraham.

Tuesday evening, February 24. Program: Deformities of the female pelvis, P. W. Van Peyma.

*Section on Surgery.*—Tuesday evening, February 3. Program: Poisonings by corrosives, Eli H. Long. Bone aneurism, Harvey R. Gaylord.

*Section on Medicine.*—Tuesday evening, February 10. Program: Hodgkin's disease, DeWitt H. Sherman, Harvey R. Gaylord. Stimulation, Eli H. Long.

*Section on Pathology.*—Tuesday evening, February 17. Program: Pathological indications for the induction of premature labor and accouchement forcé, and the consideration of the most available methods of bringing them about, J. Whitridge Williams, Professor of Obstetrics, Johns Hopkins University, Baltimore.

---

The American Medico-Psychological Association having become affiliated with the Congress of American Physicians and Surgeons, it is obligatory under the constitution and bylaws of the congress that the association hold its meeting in 1903 and every third year at Washington.. The congress has therefore instructed the secretary to issue a notice, changing the place of meeting from Providence to Washington, and fixing the dates, May 12, 13, 14 and 15, 1903.

---

THE Medical Society of the Missouri Valley will hold its Spring meeting at Council Bluffs, Iowa, on Thursday and Friday, March 19 and 20, 1903. The membership of the society includes the representative men of Iowa, Nebraska, Missouri, Kansas, North and South Dakota, and the meetings are always interesting and profitable to those who attend. A feature of the first day's session will be a symposium on syphilis, and on the second day a symposium on Typhoid fever will be presented. A cordial invitation is extended to the profession. Dr. J. M. Barstow, of Council Bluffs, Ia., is the president, and Dr. Chas. Wood Fassett, of St. Joseph, Mo., is the Secretary.

